# Robust quantum control using smooth pulses and topological winding

**DOI:** 10.1038/srep12685

**Published:** 2015-08-04

**Authors:** Edwin Barnes, Xin Wang, S. Das Sarma

**Affiliations:** 1 Condensed Matter Theory Center, Department of Physics, University of Maryland, College Park, MD 20742, USA; 2 Joint Quantum Institute, University of Maryland, College Park, MD 20742, USA; 3 Department of Physics and Materials Science, City University of Hong Kong, Kowloon, Hong Kong SAR, China

## Abstract

The greatest challenge in achieving the high level of control needed for future technologies based on coherent quantum systems is the decoherence induced by the environment. Here, we present an analytical approach that yields explicit constraints on the driving field which are necessary and sufficient to ensure that the leading-order noise-induced errors in a qubit’s evolution cancel exactly. We derive constraints for two of the most common types of noise that arise in qubits: slow fluctuations of the qubit energy splitting and fluctuations in the driving field itself. By theoretically recasting a phase in the qubit’s wavefunction as a topological winding number, we can satisfy the noise-cancelation conditions by adjusting driving field parameters without altering the target state or quantum evolution. We demonstrate our method by constructing robust quantum gates for two types of spin qubit: phosphorous donors in silicon and nitrogen-vacancy centers in diamond.

Quantum-based devices are anticipated to serve as the foundation for a new wave of technology capable of performing tasks far beyond the reach of present day electronics. At the heart of this expectation is the demand for microscopic quantum systems that can be reliably manufactured, isolated from their environment, and controlled with very high precision. Residual effects from the environment are of course inevitable, especially for solid state devices, where decoherence stems from a variety of sources, including charge noise^1^,^2^, nuclear spin fluctuations^2^,^3^,^4^, stray magnetic fields^3^, quasiparticle poisoning^5^, etc. Some level of environmental disturbance is acceptable provided these effects are not so strong as to destroy the coherence of the system before it has completed its task^6^. It has been known for several decades that a crucial ingredient in achieving this tolerance threshold is the use of carefully designed control protocols capable of dynamically correcting for the effects of noise^7^,^8^,^9^,^10^. Such methods are particularly effective in the case of a non-Markovian environment that induces fluctuations in system properties that are slow compared to the control timescales.

The search for robust control fields has been carried out over the last several decades, originating in the field of NMR and branching into newer fields such as quantum computing and nanoscale devices. Dynamical control techniques for preserving the state of an idle two-level system coupled to an environmental bath (e.g. spin echo^7^) have in fact been known for more than 60 years. In the context of quantum computing, considerable progress has been made in recent years in developing more sophisticated control protocols that extend the lifetime of a quantum state, an important step toward constructing quantum memory resources^11^,^12^,^13^. However, for the purposes of quantum information processing, it is also necessary to correct errors while a computation is being performed; this is a much more challenging objective, and it is our goal in this work. Several approaches have been pursued previously to create controls that execute a desired quantum evolution while simultaneously combatting noise using either numerical or analytical methods^4^,^14^,^15^,^16^,^17^,^18^,^19^,^20^,^21^,^22^,^23^. Numerical methods cannot easily distinguish local and global extrema in a cost function and can be difficult to use depending on the number and nature of physical constraints present in a system of interest^16^,^17^. Analytical methods suffer from the problem that very few analytical solutions to the time-dependent Schrödinger equation are known^24^, a fact which leads to proposals involving sequences of idealized control pulses, such as delta functions or square waveforms, that are often neither optimal nor easily implemented in real experimental setups. Replacing such waveforms with smoother shapes such as Gaussians^21^ can make them easier to generate in real systems, but the fact remains that using a preselected pulse shape repeatedly provides few tunable parameters and leads to unnecessarily long control sequences which may be impractical depending on the physical system in question.

In this work, we present a general solution to the quantum control problem for a two-level system. We develop an analytical approach to constructing robust dynamical control protocols that yields an unlimited number of smoothly varying, experimentally feasible driving fields for a given task. Unlike previous analytical methods, we do not preselect a particular waveform to serve as the building block of composite sequences, but rather develop a formalism that systematically generates optimal waveforms. We consider a two-level Hamiltonian of the following form:


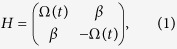


where *β* can be thought of as the qubit energy splitting and Ω(*t*) is the driving field. This Hamiltonian describes several types of qubit. For example, in the case of singlet-triplet spin qubits^1^,^2^,^3^,^25^,^26^ Ω(*t*) represents a time-dependent exchange coupling between two electron spins, and *β* is a magnetic field gradient. On the other hand, for spin qubits driven by monochromatic laser or microwave fields, Eq. (1) is the Hamiltonian in the rotating frame of the driving field, with Ω(*t*) determined by its power, and *β* is its detuning relative to the qubit’s resonance frequency^27^,^28^,^29^,^30^. Interactions between the qubit and a non-Markovian environment can induce slow fluctuations in both Ω(*t*) and *β* such that *β* = *β*_0_ + *δβ* and Ω(*t*) = Ω_0_(*t*) + *g*(*t*)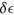, where *δβ* and 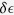 are unknown stochastic variations that are independent of each other and constant during the application of Ω(*t*), and *g*(*t*) is a function which generally depends on Ω_0_(*t*) and on the nature of the noise. Our goal is to choose a form for Ω_0_(*t*) such that the evolution operator *U*(*t*) generated by Eq. (1) achieves a target value *U*(*t*_*f*_) at some time *t*_*f*_ and where *U*(*t*_*f*_) is independent of *δβ* and 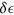 to first order in these fluctuations. While finding all possible driving fields Ω_0_(*t*) that achieve this may seem like an impossible task, we show that it proves to be surprisingly tractable. Below, we obtain a general solution to this problem by deriving a set of constraints which any robust control field must obey and showing how these can be solved systematically.

The starting point of our method is a recently proposed formalism for generating forms of Ω(*t*) for which the corresponding Schrödinger equation can be solved exactly^24^,^31^,^32^. The basic idea is to parameterize both the driving field Ω(*t*) and the evolution operator *U*(*t*) in terms of a single function denoted by *χ*(*t*). In particular, the driving field can be expressed as


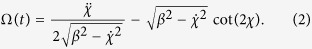


A similar expression for *U*(*t*) in terms of *χ*(*t*) along with a brief description of the formalism can be found in the Supplementary Information. The main result of Ref. [31] is that any choice of *χ*(*t*) obeying the inequality 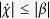 yields an analytical expression for the evolution *U*(*t*) generated by the Ω(*t*) determined from Eq. (2), where the inequality enforces the unitarity of *U*(*t*).

In the present context of designing robust controls, the utility of the *χ*(*t*) formalism is that it allows us to trace how fluctuations in the Hamiltonian give rise to fluctuations in the evolution operator. In particular, the fluctuations *δβ* and 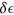 will induce time-dependent fluctuations of *χ*: 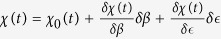. In the Supplementary Information, we show that, remarkably, the fluctuations in *χ*(*t*) can be calculated exactly analytically:


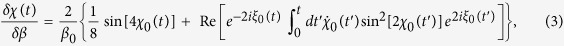



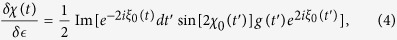


where 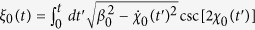 is a phase appearing in *U*(*t*). Since the evolution *U*(*t*) is a functional of *χ*(*t*), we can use Eqs. (3) and (4) to derive the corresponding variations of *U*(*t*) due to noise. We can then construct noise-resistant driving fields by requiring these variations to vanish at the final time *t* = *t*_*f*_; this imposes constraints on *χ*_0_(*t*), the solutions to which can then be input into Eq. (2) to obtain forms of Ω_0_(*t*) that implement robust control.

A challenge of the strategy we have just outlined is that it does not include a means of fixing the net evolution *U*(*t*_*f*_) to a target value. For example, we could solve the constraints on *χ*_0_(*t*) by picking an ansatz for this function that includes free parameters that can be adjusted until the constraints are satisfied. As we tune these parameters, though, we must also ensure that *U*(*t*_*f*_) does not vary, and this is made difficult by the presence of the phase *ξ*_0_(*t*) in *U*(*t*); the fact that this phase is an integral of a complicated nonlinear expression involving *χ*_0_(*t*) makes it challenging to hold *ξ*_0_(*t*_*f*_) fixed as parameters in *χ*_0_(*t*) are varied.

We circumvent this formidable problem by observing that the invariance of *ξ*_0_(*t*_*f*_) under parameter variations is tantamount to saying that this phase is a topological winding number. In this point of view, *ξ*_0_(*t*) is proportional to the phase of a complex function which traces a contour in the complex plane that winds one or more times around the origin as time evolves from *t* = 0 to *t* = *t*_*f*_. Changing parameters in *χ*_0_(*t*) deforms this contour but preserves the winding number provided the contour does not cross the origin. Thus, the quantization of this topological winding number enables us to fix the target evolution while eliminating leading-order unknown errors in the qubit evolution.

In practice, we implement this idea by expressing the phase *ξ*_0_(*t*) as a functional of *χ*_0_(*t*): *ξ*_0_(*t*) = Φ[*χ*_0_(*t*)]. This allows us to control the final value of the phase *ξ*_0_(*t*_*f*_) directly from the function Φ(*χ*) without fixing *ξ*_0_(*t*) itself, which would be far too restrictive. By equating the integrand of *ξ*_0_(*t*) to 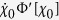, we see that we can reproduce *χ*_0_(*t*) from Φ(*χ*) through the formula


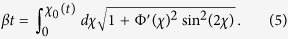


Thus, for a given Φ(*χ*), we can obtain the driving field Ω_0_(*t*) and complete time-dependence of the evolution operator by first performing the integral in Eq. (5) and inverting the result to find *χ*_0_(*t*). Moreover, as shown in the Supplementary Information, we can convert the noise-cancelation constraints derived earlier for *χ*_0_(*t*) into a general set of constraints on Φ(*χ*). For example, in the case of time-antisymmetric driving fields (Ω(−*t*) = −Ω(*t*) for controls applied from *t* = −*t*_*f*_ to *t* = *t*_*f*_), the constraints for canceling *δβ*-noise and 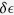-noise are respectively


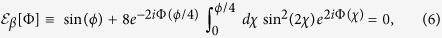



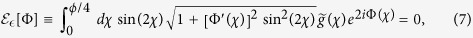


where 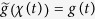, and *ϕ* is the target rotation angle. We can visualize the solution space of these constraints by choosing an ansatz for Φ(*χ*) that contains adjustable parameters and then plotting 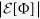 as a function of these parameters. An example of such an “error potential” is shown in Fig. 1(a) for an ansatz containing two free parameters. The points in parameter space where the error potential vanishes yield driving fields that implement robust quantum control. One additional constraint for each type of noise must be satisfied by Φ(*χ*) for more general driving fields (see Supplementary Information). If Φ(*χ*) satisfies both (6) and (7), the corresponding evolution will be immune to both types of error. One can also suppress pulse timing errors by imposing constraints on the initial and final values of the higher derivatives of Φ(*χ*), which produces a flattening of the tails of the pulse (see Supplementary Information).

The expression on the right-hand side of Eq. (5) can be graphically interpreted as the length of a curve lying on the surface of a sphere parameterized by polar angle *χ*/2 and azimuthal angle Φ/2. This reveals an underlying geometrical picture in which the driving field is represented as a string on the sphere’s surface which extends from the north pole (*χ* = 0) down to a point *χ* = *ϕ*/4, Φ = *ξ*_0_(*t*_*f*_) determined by the target evolution *U*(*t*_*f*_). Examples are shown in Fig. 1(c), with the corresponding driving fields displayed in Fig. 1(d–f). As illustrated in Fig. 1, the noise-cancelation constraints generally admit multiple solutions, translating to a collection of different strings which all start and end at the same points. Eq. (5) indicates that the total duration of the control field is given by the length of the string: 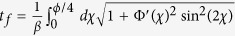. This observation shows that functions Φ(*χ*) which minimize this expression yield the control fields that generate the fastest possible target evolutions. In the context of robust quantum control, this minimization should be performed over the set of solutions to the noise-cancelation constraints.

We demonstrate our method by applying it to two types of solid state qubits which are currently at the forefront of quantum technology research. The first type of qubit is comprised of the two spin states of an electron confined to a phosphorous donor in silicon^29^,^33^,^34^,^35^,^36^, where one of the primary manifestations of noise stems from power fluctuations in the waveform generators used to implement the control fields^29^,^37^. Provided that these power fluctuations are slow compared to the duration of the applied field, we can model this as 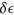-noise where the function *g*(*t*) characterizing the noise is proportional to the intended field Ω_0_(*t*). We use the ansatz for Φ(*χ*) given in Fig. 1 which yields rotations about a particular axis in the *xy* plane (see Supplementary Information for a universal set of robust quantum gates). To demonstrate the cancelation of noise, we show the infidelity as a function of the noise strength in Fig. 2. For comparison, we have also included the infidelity incurred by an ordinary piecewise square pulse that implements the same rotation. The orders of magnitude reduction in the infidelity and the change in slope of the curve clearly demonstrate the cancelation of the leading-order errors in the target evolution.

The second example we consider is a spin qubit in a nitrogen-vacancy center in diamond^4^,^30^,^38^. In this case, a leading source of noise is fluctuations in the qubit energy splitting due to hyperfine interactions with neighboring nuclear spins^4^,^30^. These fluctuations are typically very slow and naturally modeled in terms of *δβ*-noise. We can again use an ansatz like that given in Fig. 1 and tune parameters to satisfy the noise-cancelation condition. Details along with parameters for a complete set of universal gates are given in the Supplementary Information. Here, we note that 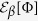 can also be made to vanish exactly when *ϕ* = *nπ* for some integer *n* by choosing 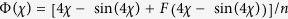, where *F*(*θ* + *nπ*/2) = *F*(*θ*) is any periodic function with period *nπ*/2. One of the corresponding driving fields which produces a *π* rotation about an axis in the *xy* plane is shown in Fig. 3(a). A striking reduction in noise relative to the performance of a generic control field (see Fig. 3(b) is revealed in a comparison of the respective infidelities, shown in Fig. 3(c).

The results presented here show that analytical methods based on a deep theoretical analysis of qubit dynamics can be a powerful tool in developing experimentally feasible robust quantum controls involving the application of smooth practical external pulses. Future work will include further optimization in terms of minimizing the control durations, including additional driving terms in the Hamiltonian, allowing for non-static noise with a well-defined power spectrum, and extending the approach to multi-level quantum systems. We anticipate that such methods will play an important role in overcoming the decoherence problem in microscopic quantum systems. In particular, we believe that the theoretical techniques presented here will be essential in reducing errors in solid state quantum computing architectures down to the level of the quantum error correction threshold so that scalable quantum information processing may become feasible in the laboratory.

## Additional Information

**How to cite this article**: Barnes, E. *et al.* Robust quantum control using smooth pulses and topological winding. *Sci. Rep.* **5**, 12685; doi: 10.1038/srep12685 (2015).

## Supplementary Material

Supplementary Information

## Figures and Tables

**Figure 1:**
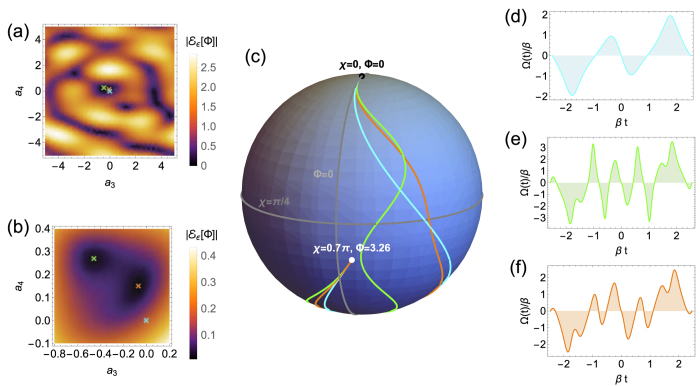
(**a**) The error potential associated with 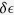-noise with *g*(*t*) = Ω_0_(*t*) for an ansatz of the form 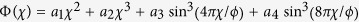 with *ϕ* = 2.8*π*, *a*_1_ = 0.74, *a*_2_ = −0.18, corresponding to a rotation about the axis 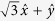 in the *xy* plane. The parameter regions where the first-order error in the evolution operator vanishes are shown in black. (**b**) A zoom-in on the central region of (**a**) revealing two points where the error vanishes (marked with green and orange). (**c**) Geometrical representation of control fields as curves on the surface of a sphere which extend from the north pole to a final point determined by the target evolution. The green and orange curves correspond to the two points of vanishing error shown in (**b**), while the cyan curve corresponds to the point *a*_3_ = *a*_4_ = 0 at which the error is nonzero. The lengths of the curves give the durations of the respective control fields. (**d–f**) The control fields for each of the curves shown in (**c**). Each control field implements the same rotation in approximately the same time. The driving fields shown in (**e**) and (**f**) dynamically cancel the 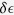 error, while that shown in (**d**) does not.

**Figure 2:**
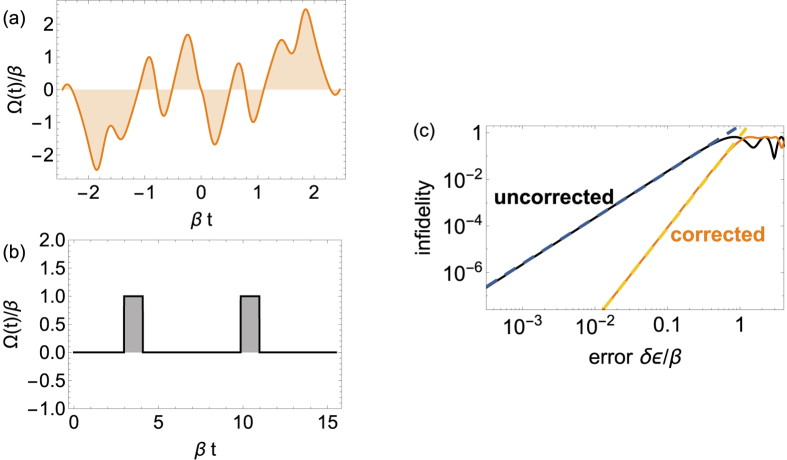
(**a**) Pulse from Fig. 1(f) designed to self-correct for driving field errors (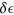-errors). This pulse implements a rotation about the axis 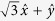 by angle 2.8*π*. In the context of electron spin qubits in silicon, Ω(*t*) is the envelope and *β* is the detuning of an external microwave field. Typical microwave generators produce waveforms obeying 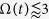 MHz^29^, in which case we should choose *β* ≈ 1 MHz, yielding a 5 *μ*s pulse. (**b**) An ordinary pulse that implements the same rotation as the pulse in (**a**) but which does not satisfy the error-cancelation constraint in Eq. (7). (**c**) Comparison of the infidelities incurred by the control fields of (**a**) and (**b**). The orders of magnitude reduction in the infidelity and the change in the slope of the corrected curve relative to the uncorrected demonstrate first-order error cancelation. Blue and orange dashed lines are quadratic and quartic fits, respectively: 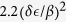, 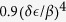.

**Figure 3:**
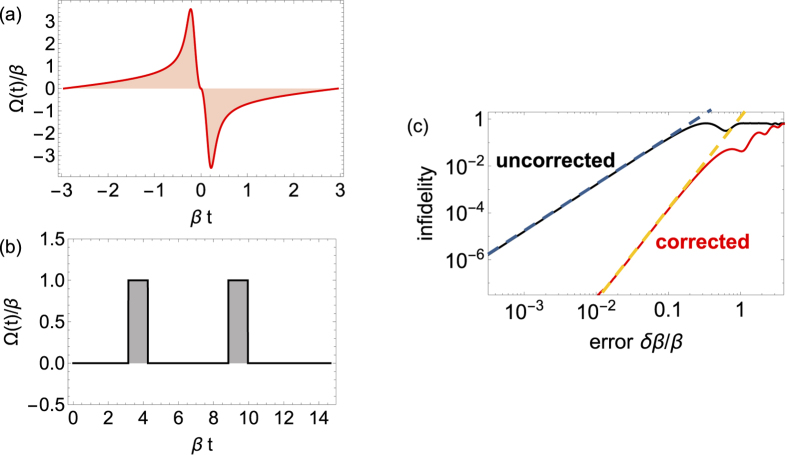
(**a**) Driving field derived from choosing Φ(*χ*) = 4*χ* − sin(4*χ*). This pulse implements a *π* rotation about the axis 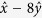 while canceling *δβ*-noise, which represents hyperfine noise in the case of NV centers in diamond. In this context, we can interpret Ω(*t*) as the amplitude of a microwave pulse with detuning *β*. For a pulse of maximum amplitude 20 MHz, choosing *β* = 5 MHz leads to a pulse duration of 1.2 *μ*s. (**b**) An ordinary pulse that implements the same rotation as (**a**) but without built-in error suppression. (**c**) A comparison of the infidelities incurred by the driving fields shown in (**a**) and (**b**) exhibits the noise cancelation effected by pulse (**a**). Blue and orange dashed lines are quadratic and quartic fits, respectively: 15(*δβ*/*β*)^2^, 1.5(*δβ*/*β*)^4^.
