# Supplementary material for: Robust quantum control using smooth pulses and topological winding
Source: Sci Rep. 2015 Aug 4;5:12685. doi: 10.1038/srep12685 (PMC4523836; doi:10.1038/srep12685)
Supplement: Supplementary Information [file srep12685-s1.pdf]

Robust quantum control using smooth pulses and topological winding: Supplementary Information

Edwin Barnes[1,2], Xin Wang[1], & S. Das Sarma[1,2]
[1]*Condensed Matter Theory Center, Department of Physics, University of Maryland, College Park, MD 20742, USA*
[2]*Joint Quantum Institute, University of Maryland, College Park, MD 20742, USA*

I. ANALYTICALLY SOLVABLE DRIVING FIELDS

As shown in Ref. [1], the dynamical evolution operator for a general, time-dependent two-level quantum system described by the Hamiltonian

$$H = b_x(t)\sigma_x + b_y(t)\sigma_y + b_z(t)\sigma_z, \tag{1}$$

can be written in the form

$$U = \begin{pmatrix} u_{11} & -u_{21}^* \\ u_{21} & u_{11}^* \end{pmatrix}, \qquad |u_{11}|^2 + |u_{21}|^2 = 1, \tag{2}$$

where the explicit u_{11}, u_{21} and driving fields are

$$\begin{aligned} u_{11} &= \cos\chi e^{i\xi_- - i\varphi/2}, \quad u_{21} = i\eta \sin\chi e^{i\xi_+ + i\varphi/2}, \\ \xi_\pm &= \int_0^t dt' \beta \sqrt{1 - \frac{\dot\chi^2}{\beta^2}} \csc(2\chi) \pm \frac{1}{2}\arcsin\left(\frac{\dot\chi}{\beta}\right) \pm \eta\frac{\pi}{4}, \\ b_x &= \beta\cos\varphi, \quad b_y = \beta\sin\varphi, \\ b_z &= \frac{\ddot\chi - \dot\chi\dot\beta/\beta}{2\beta\sqrt{1-\dot\chi^2/\beta^2}} - \beta\sqrt{1-\dot\chi^2/\beta^2}\cot(2\chi) + \frac{\dot\varphi}{2}. \end{aligned} \tag{3, 4}$$

$\beta(t)$, $\varphi(t)$, and $\chi(t)$ are three auxiliary functions that allow us to more easily determine how the properties of H influence the behavior of U. The main result of Ref. [1] is that any solution to the time-dependent Schrödinger equation corresponds to some choice of these three auxiliary functions for which the inequality $|\dot\chi| \le |\beta|$ is satisfied. The initial conditions $u_{11}(0)=1$, $u_{21}(0)=0$ imply $\chi(0)=0$, and $\dot\chi(0)=-\eta\beta(0)$ ensures $b_z(0)$ is finite, where $\eta=\pm1$. In the most general case of driving along all three axes, all three auxiliary functions have nontrivial time dependence. In this work, we are only interested in the case of single-axis driving, for which we have $\varphi = 0$ and β is a constant. In this case, the Hamiltonian reduces to the form

$$H = \beta\sigma_x + \Omega(t)\sigma_z, \tag{5}$$

where we have defined $b_z(t) = \Omega(t)$. This Hamiltonian is in a form that is relevant for several types of experimentally relevant two-level quantum systems, for example singlet-triplet spin qubits in semiconductor quantum dots.[2]

In many types of qubits, particularly those used in electron spin resonance or nuclear magnetic resonance experiments, the effective qubit Hamiltonian can be expressed as

$$H_{qubit} = \begin{pmatrix} E/2 & \Omega(t)e^{-i\omega t} \\ \Omega(t)e^{i\omega t} & -E/2 \end{pmatrix}, \tag{6}$$

where E is the qubit energy splitting and $\Omega(t)e^{i\omega t}$ represents a monochromatic pulse with envelope Ω and frequency ω. The simplest way to adapt our formalism to this case is to first perform the following transformation to the rotating frame:

$$T = e^{-i\frac{\omega t}{2}\sigma_z} e^{-i\frac{\pi}{4}\sigma_y}, \tag{7}$$

yielding

$$H_{rot} = \begin{pmatrix} \Omega(t) & (\omega - E)/2 \\ (\omega - E)/2 & -\Omega(t) \end{pmatrix}. \tag{8}$$

This rotating-frame Hamiltonian has the form of Eq. (5) with $\beta = (\omega - E)/2$. In the context of NV centers or electron spin qubits in phosphorous donors in silicon, fluctuations in β result from fluctuations in E, which in turn are caused by Overhauser noise arising from nuclear spins in the environment. In both these contexts, as well as in the case of nuclear spin qubits in phosphorous donors, noise in $\Omega(t)$ would result from e.g., power fluctuations in the pulse generator. Such fluctuations were identified as a possible cause of nuclear spin control infidelities.[3]

Although H_{rot} has the same form as Eq. (5), it is important to note that we want the evolution operator in the lab frame (i.e., the evolution operator associated with H_{qubit} above) to be the identity matrix at $t = 0$. The evolution operators corresponding to H_{qubit} and H_{rot} are related by

$$U_{lab} = TU_{rot}, \tag{9}$$

so that our initial condition for U_{rot} should then be

$$U_{rot}(0) = T^{\dagger}(0) = e^{i\frac{\pi}{4}\sigma_y} = \frac{1}{\sqrt{2}}\begin{pmatrix} 1 & 1 \\ -1 & 1 \end{pmatrix}. \tag{10}$$

One way to incorporate this new initial condition would be to simply multiply U_{rot} on the right by $T^{\dagger}(0)$. This will re-arrange the components of the evolution operator shown in Eq. (3) but does not modify the error cancelation procedure we will develop using the Hamiltonian given in Eq. (5).

II. FLUCTUATIONS IN β

In this section, we want to consider the situation where the qubit splitting (or external field detuning) β exhibits stochastic fluctuations that are constant throughout the duration of the pulse, i.e., $\beta = \beta_0 + \delta\beta$, where β_0 is a known constant and $\delta\beta$ is an unknown constant. We would like to engineer pulses that execute a target evolution while minimizing the errors caused by the unknown variation $\delta\beta$. In order to set up this problem, it turns out to be beneficial to use a new parametrization of the driving field and the corresponding evolution operator. We first derive this new parametrization and then return to the question of how to incorporate stochastic fluctuations into our formalism for generating analytical solutions of two-level quantum dynamics.

The first step in deriving the new parametrization is to transform to a rotating frame in the x-basis:

$$D_{\pm} = \frac{1}{\sqrt{2}}e^{\pm i\beta t}(u_{11} \pm u_{21}). \tag{11}$$

The functions $D_{\pm}$ then solve the following set of equations which follow from the Schrödinger equation for the evolution operator U:

$$\dot{D}_{\pm} = -i\Omega e^{\pm 2i\beta t} D_{\mp}. \tag{12}$$

These two equations can be solved easily for $\Omega(t)$:

$$\Omega^2 = -\frac{\dot{D}_+\dot{D}_-}{D_+D_-}, \tag{13}$$

which implies that we may write

$$\frac{\dot{D}_+}{D_+} = -i\Omega/u, \quad \frac{\dot{D}_-}{D_-} = -i\Omega u, \tag{14}$$

where $u = u(t, \beta)$ is an unknown complex function. These two equations are easily solved:

$$\begin{aligned} D_+ &= \frac{1}{\sqrt{2}}e^{-i\int_0^t dt'\Omega(t')/u(t',\beta)}, \\ D_- &= \frac{1}{\sqrt{2}}e^{-i\int_0^t dt'\Omega(t')u(t',\beta)}, \end{aligned} \tag{15}$$

where we have imposed the initial conditions $D_{\pm}(0) = 1/\sqrt{2}$.

Equations (12) also imply

$$\dot{D}_+D_+e^{-2i\beta t} = \dot{D}_-D_-e^{2i\beta t}. \tag{16}$$

Plugging Eqs. (15) into this equation yields

$$e^{2i\int_0^t dt'\Omega(t')[u(t',\beta)-1/u(t',\beta)]} = u^2(t,\beta)e^{4i\beta t}. \tag{17}$$

Differentiating both sides of this equation with respect to time and performing some algebraic manipulations results in

$$\Omega = -i\frac{\dot{u}+2i\beta u}{u^2-1}. \tag{18}$$

Writing

$$u(t,\beta) = \tanh[w(t,\beta)], \tag{19}$$

we have

$$\Omega(t) = i\dot{w} - \beta\sinh(2w). \tag{20}$$

It is straightforward to express $D_\pm$ in terms of w:

$$\begin{aligned} D_+ &= \frac{1}{\sqrt{2}}\sinh(w)e^{2i\beta\int_0^t dt'\cosh^2(w(t'))}, \\ D_- &= \frac{1}{\sqrt{2}}\cosh(w)e^{2i\beta\int_0^t dt'\sinh^2(w(t'))}. \end{aligned} \tag{21}$$

Any choice of $w(t)$ such that the $\Omega(t)$ computed from Eq. (20) is real immediately yields an analytical solution to the Schrödinger equation. It is straightforward to find the necessary restriction on w. Writing $w = w_r + iw_i$ in Eq. (20), the condition $\mathrm{Im}[\Omega] = 0$ implies

$$\dot{w}_r = \beta\sin(2w_i)\cosh(2w_r). \tag{22}$$

This equation is easily integrated to give

$$w_r(t) = \frac{1}{2}\log\tan\left[\beta\int_0^t \sin(2w_i(t')) + c\right]. \tag{23}$$

In this expression, c is an integration constant determined by the boundary condition $w_r(0) = \frac{1}{2}\log\tan c$, which we will leave arbitrary, at least for now. We must impose

$$0 \le 2\beta\int_0^t dt'\sin(2w_i(t')) + 2c \le \pi \tag{24}$$

to ensure that w_r is real. Plugging Eq. (23) into Eq. (20), we find an expression for Ω in terms of w_i:

$$\Omega(t) = -\dot{w}_i + \beta\cos(2w_i)\cot\left[2\beta\int_0^t dt'\sin(2w_i(t')) + 2c\right]. \tag{25}$$

This result can in fact be obtained from the method given in Ref. [4] by choosing

$$q(t) = \cos\left[2\beta\int_0^t dt'\sin(2w_i(t')) + 2c\right]. \tag{26}$$

So far, we have derived an alternate algorithm for generating analytical solutions to the Schrödinger equation: An analytical solution can be produced by choosing any $w_i(t)$ such that $0 \le 2\beta\int_0^t dt'\sin(2w_i(t')) + 2c \le \pi$.

We now consider the case where $\beta = \beta_0 + \delta\beta$, where β_0 is a known constant and $\delta\beta$ is a small stochastic variation. Since our formula for $\Omega(t)$, Eq. (20), contains an explicit dependence on β, we must take care to choose $w(t)$ in such a way that the resulting $\Omega(t)$ obeys $\Omega(t,\beta) = \Omega(t,\beta_0) + \mathcal{O}(\delta\beta^2)$. This condition is necessary since $\Omega(t)$ cannot itself depend on the stochastic variable $\delta\beta$. As long as we are only concerned with first-order variations in $\delta\beta$, it is sufficient to require the first-order variation of $\Omega(t)$ to vanish. Note that the present analysis could be extended to derive constraints which ensure that higher-order errors in the evolution operator vanish by requiring the corresponding higher-order variations of $\Omega(t)$ to vanish.

We can arrange for the first-order variation of $\Omega(t)$ to vanish by writing $w = w_0 + \delta\beta w_1$ and varying Eq. (20) to obtain

$$\Omega(t) = i\dot{w}_0 - \beta_0 \sinh(2w_0) + [i\dot{w}_1 - \sinh(2w_0) - 2\beta_0 w_1 \cosh(2w_0)]\,\delta\beta + \mathcal{O}(\delta\beta^2). \tag{27}$$

The first-order variation will vanish if w_1 is given by

$$w_1(t) = e^{-2i\beta_0 \int_0^t dt' \cosh(2w_0(t'))}\left[k - i\int_0^t dt' e^{2i\beta_0 \int_0^{t'} dt'' \cosh(2w_0(t''))} \sinh(2w_0(t'))\right], \tag{28}$$

where k is an integration constant. What this result means is that we may still use our formalism for constructing analytical solutions even in the presence of the stochastic fluctuation $\delta\beta$. However, unless we arrange for higher-order variations in $\Omega(t)$ to vanish as well, we can only obtain the evolution up to first order in $\delta\beta$. In most physical situations, $\delta\beta \ll \beta_0$, and determining the evolution up to first order in $\delta\beta$ is sufficient. The procedure works by first choosing $w_{0,i}$ to fix the desired driving field, $\Omega(t)$. Choosing $w_{0,i}$ also fixes $w_{0,r}$ and w_0, and the latter then determines w_1. The corresponding evolution operator to first order in $\delta\beta$ is obtained by using $w = w_0 + \delta\beta w_1$ in Eq. (21).

We have just seen that the w-parametrization is useful for removing the $\delta\beta$ dependence from $\Omega(t)$. However, now that we have laid out the necessary steps, it turns out that further simplifications occur if we return to the χ-parametrization. In particular, we may simplify the expression for w_1. To see this, begin with the general relation between the two parameterizations:

$$\chi(t) = \beta \int_0^t dt' \sin(2w_i(t')) + c. \tag{29}$$

Since we wish to impose $\chi(0) = 0$, we will set $c = 0$ from now on. Using this expression for $\chi(t)$, it is straightforward to show

$$w_0 = \tfrac{1}{2}\log\tan\chi_0 + i\tfrac{1}{2}\sin^{-1}\left(\frac{\dot{\chi}_0}{\beta_0}\right), \tag{30}$$

so that

$$\begin{aligned}\cosh(2w_0) &= \csc(2\chi_0)\sqrt{1 - \frac{\dot{\chi}_0^2}{\beta_0^2}} - i\cot(2\chi_0)\frac{\dot{\chi}_0}{\beta_0},\\ \sinh(2w_0) &= -\cot(2\chi_0)\sqrt{1 - \frac{\dot{\chi}_0^2}{\beta_0^2}} + i\csc(2\chi_0)\frac{\dot{\chi}_0}{\beta_0},\end{aligned} \tag{31}$$

where

$$\chi_0(t) = \beta_0 \int_0^t dt' \sin(2w_{i,0}(t')). \tag{32}$$

Comparing this result for $\cosh(2w_0)$ with the definition of the function $\xi(t)$ defined earlier, we see that

$$2\beta_0 \int_0^t dt' \cosh(2w_0(t')) = 2\xi_0(t) - i\log\sin[2\chi_0(t)], \tag{33}$$

where here $\xi_0(t)$ is defined in terms of $\chi_0(t)$ (and not the full $\chi(t)$):

$$\xi_0(t) = \int_0^t dt' \sqrt{\beta^2 - \dot{\chi}_0(t')^2}\csc[2\chi_0(t')]. \tag{34}$$

Substituting this result into Eq. (28) and setting the integration constant k therein to zero then yields

$$\begin{aligned}
w_1(t) &= -ie^{-2i\xi_0(t)}\csc[2\chi_0(t)]\int_0^t dt' e^{2i\xi_0(t')}\sin[2\chi_0(t')]\left\{-\cot[2\chi_0(t')]\sqrt{1-\frac{\dot\chi_0^2(t')}{\beta_0^2}}+i\csc[2\chi_0(t')]\frac{\dot\chi_0(t')}{\beta_0}\right\} \\
&= -\frac{i}{\beta_0}e^{-2i\xi_0(t)}\csc[2\chi_0(t)]\int_0^t dt' e^{2i\xi_0(t')}\left\{-\tfrac{1}{2}\dot\xi_0(t')\sin[4\chi_0(t')]+i\dot\chi_0(t')\right\} \\
&= -\frac{i}{\beta_0}e^{-2i\xi_0(t)}\csc[2\chi_0(t)]\int_0^t dt'\left\{\frac{d}{dt'}\left[\frac{i}{4}e^{2i\xi_0(t')}\sin[4\chi_0(t')]\right]-\frac{i}{4}e^{2i\xi_0(t')}\frac{d}{dt'}\sin[4\chi_0(t')]+i\dot\chi_0(t')e^{2i\xi_0(t')}\right\} \\
&= -\frac{i}{\beta_0}e^{-2i\xi_0(t)}\csc[2\chi_0(t)]\left\{\frac{i}{4}e^{2i\xi_0(t)}\sin[4\chi_0(t)]+2i\int_0^t dt'\dot\chi_0(t')e^{2i\xi_0(t')}\sin^2[2\chi_0(t')]\right\} \\
&= \frac{1}{2\beta_0}\cos[2\chi_0(t)]+\frac{2}{\beta_0}e^{-2i\xi_0(t)}\csc[2\chi_0(t)]\int_0^t dt'\dot\chi_0(t')e^{2i\xi_0(t')}\sin^2[2\chi_0(t')]. \qquad (35)
\end{aligned}$$

The first-order variation, $\delta w = \delta\beta w_1$, leads to the following variation in $\chi(t)$:

$$\begin{aligned}
\delta_\beta\chi(t) &= \delta\beta\frac{\chi_0(t)}{\beta_0}+2\delta\beta\int_0^t dt'\sqrt{\beta_0^2-\dot\chi_0^2(t')}\,\mathrm{Im}[w_1(t')] \\
&= \delta\beta\frac{\chi_0(t)}{\beta_0}+2\delta\beta\int_0^t dt'\dot\xi_0(t')\sin[2\chi_0(t')]\mathrm{Im}[w_1(t')] \\
&= \delta\beta\frac{\chi_0(t)}{\beta_0}+\delta\beta\frac{4}{\beta_0}\int_0^t dt'\dot\xi_0(t')\Bigg\{\cos[2\xi_0(t')]\int_0^{t'} dt''\dot\chi_0(t'')\sin^2[2\chi_0(t'')]\sin[2\xi_0(t'')] \\
&\quad -\sin[2\xi_0(t')]\int_0^{t'} dt''\dot\chi_0(t'')\sin^2[2\chi_0(t'')]\cos[2\xi_0(t'')]\Bigg\} \\
&= \delta\beta\frac{\chi_0(t)}{\beta_0}+\delta\beta\frac{2}{\beta_0}\Bigg\{\sin[2\xi_0(t)]\int_0^t dt'\dot\chi_0(t')\sin^2[2\chi_0(t')]\sin[2\xi_0(t')] \\
&\quad +\cos[2\xi_0(t)]\int_0^t dt'\dot\chi(t')\sin^2[2\chi_0(t')]\cos[2\xi_0(t')]\Bigg\} \\
&\quad -\delta\beta\frac{2}{\beta_0}\int_0^t dt'\left(\sin^2[2\xi_0(t')]\sin^2[2\chi_0(t')]+\cos^2[2\xi_0(t')]\sin^2[2\chi_0(t')]\right)\dot\chi_0(t') \\
&= \delta\beta\frac{2}{\beta_0}\Bigg\{\frac{1}{8}\sin[4\chi_0(t)]+\sin[2\xi_0(t)]\int_0^t dt'\dot\chi_0(t')\sin^2[2\chi_0(t')]\sin[2\xi_0(t')] \\
&\quad +\cos[2\xi_0(t)]\int_0^t dt'\dot\chi_0(t')\sin^2[2\chi_0(t')]\cos[2\xi_0(t')]\Bigg\} \\
&= \delta\beta\frac{2}{\beta_0}\left\{\frac{1}{8}\sin[4\chi_0(t)]+\mathrm{Re}\left[e^{-2i\xi_0(t)}\int_0^t dt'\dot\chi_0(t')\sin^2[2\chi_0(t')]e^{2i\xi_0(t')}\right]\right\}. \qquad (36)
\end{aligned}$$

This completes the derivation of Eq. (3a) of the main text. Below, we will show how to use this final expression for $\delta_\beta\chi$ to construct dynamically corrected qubit operations with smooth analytical pulses.

III. FLUCTUATIONS IN $\Omega(t)$

To see how we can include first-order fluctuations of the form

$$\Omega(t)=\Omega_0(t)+\delta\epsilon\frac{d\Omega(t)}{d\epsilon}\equiv\Omega_0(t)+\delta\epsilon g(t), \qquad (37)$$

we again start from the w-parametrization from Eq. (20):

$$\Omega(t)=i\dot w(t)-\beta\sinh[2w(t)]. \qquad (38)$$

Writing $w(t)=w_0(t)+\delta\epsilon w_1(t)$, expanding the right hand side to first order in $\delta\epsilon$ and equating all the first-order terms, we find

$$g(t)=i\dot w_1-2\beta w_1\cosh(2w_0). \qquad (39)$$

This equation is easily solved, with the result

$$w_1(t) = e^{-2i\beta\int_0^t dt' \cosh[2w_0(t')]} \left[k - i\int_0^t dt' e^{2i\beta\int_0^{t'} dt'' \cosh[2w_0(t'')]} g(t')\right], \tag{40}$$

where k is an integration constant. Using Eq. (33), we can rewrite this as

$$w_1(t) = e^{-2i\xi_0(t)} \csc[2\chi_0(t)] \left[k - i\int_0^t dt' e^{2i\xi_0(t')} \sin[2\chi_0(t')] g(t')\right]. \tag{41}$$

In order to translate this result into a variation $\delta_\epsilon\chi(t)$, we use Eq. (29) which yields

$$\begin{aligned} \delta_\epsilon\chi(t) &= \delta\epsilon\beta\int_0^t dt' \mathrm{Im}[w_1(t')] \cos[2\mathrm{Im}[w_0(t')]] = \delta\epsilon\int_0^t dt' \mathrm{Im}[w_1(t')]\sqrt{\beta^2 - \dot\chi_0(t')^2} \\ &= \delta\epsilon\int_0^t dt' \mathrm{Im}[w_1(t')]\dot\xi_0(t') \sin[2\chi_0(t')]. \end{aligned} \tag{42}$$

Using that

$$\begin{aligned} \mathrm{Im}[w_1(t)] &= -\cos[2\xi_0(t)] \csc[2\chi_0(t)] \int_0^t dt' \cos[2\xi_0(t')] \sin[2\chi_0(t')] g(t') \\ &\quad - \sin[2\xi_0(t)] \csc[2\chi_0(t)] \left[k + \int_0^t dt' \sin[2\xi_0(t')] \sin[2\chi_0(t')] g(t')\right], \end{aligned} \tag{43}$$

and integrating by parts, we have

$$\begin{aligned} \delta_\epsilon\chi(t) &= \delta\epsilon\frac{1}{2}\Bigg\{ -\sin[2\xi_0(t)] \int_0^t dt' \cos[2\xi_0(t')] \sin[2\chi_0(t')] g(t') \\ &\quad + \cos[2\xi_0(t)] \left[k + \int_0^t dt' \sin[2\xi_0(t')] \sin[2\chi_0(t')] g(t')\right] \Bigg\} \\ &= \delta\epsilon\frac{1}{2}\left\{k\cos[2\xi_0(t)] + \mathrm{Im}\left[e^{-2i\xi_0(t)} \int_0^t dt' \sin[2\chi_0(t')] g(t') e^{2i\xi_0(t')}\right]\right\}. \end{aligned} \tag{44}$$

This completes the derivation of Eq. (3b) of the main text.

IV. CONTROLLED ROTATIONS VIA TOPOLOGY

The χ formalism reviewed above allows us to construct arbitrary rotations by choosing $\chi(t)$ appropriately. However, fixing the target evolution precisely remains challenging because of the fact that the phase $\xi(t) = (\xi_+ + \xi_-)/2$ appearing in the evolution operator (3) is given as an integral of a complicated nonlinear expression involving $\chi(t)$:

$$\xi(t) = \int_0^t dt' \sqrt{\beta^2 - \dot\chi^2(t')} \csc[2\chi(t')]. \tag{45}$$

Even though we get to choose the form of $\chi(t)$ with only the relatively weak constraint $|\dot\chi| \le |\beta|$ to worry about, it is difficult to predict what values of $\xi_f = \xi(t_f)$ at the final time t_f can be achieved with a given choice, and ultimately this generally requires numerical evaluation of the integral above. Moreover, if we wish to include tunable parameters in $\chi(t)$ in order to cancel errors and/or improve rotational fidelities, this task is hindered by the complicated nature of the above expression for $\xi(t)$ since it will be hard to keep ξ_f fixed as such parameters are varied. In this section, we will show how these problems can be circumvented by incorporating the concept of topological winding into our formalism.

The key observation is to notice that the invariance of ξ_f under parameter variations in $\chi(t)$ is equivalent to the statement that ξ_f is a quantized topological winding number. This quantization can be imposed by choosing $\chi(t)$ in such a way that the integrand in ξ is proportional to the derivative of the argument of a complex function $W(t)$:

$$\sqrt{\beta^2 - \dot\chi^2} \csc(2\chi) = \lambda\partial_t \arg[W(t)], \tag{46}$$

where λ is a real constant that we are free to choose. If $W(t)$ winds around the origin of the complex W plane an integral number (n) of times as time evolves from $t=0$ to $t=t_f$, then

$$\int_0^{t_f} dt\partial_t \arg[W(t)] = 2\pi n. \tag{47}$$

The value of ξ_f will then be quantized (in units of $2\pi\lambda$) according to

$$\xi_f = \int_0^{t_f} dt\sqrt{\beta^2 - \dot{\chi}^2}\csc(2\chi) = 2\pi n\lambda. \tag{48}$$

This formula shows that we can fix ξ_f to be any value we like by choosing λ appropriately and by picking a $W(t)$ which exhibits nontrivial winding. Including tunable parameters in $\chi(t)$ is tantamount to including them in $W(t)$; adjusting these parameters will deform the contour traced by $W(t)$ but will leave the winding number n the same so long as the contour does not cross the origin in the process. Once $W(t)$ is chosen, we then solve Eq. (46) to obtain $\chi(t)$, from which the corresponding driving field, $\Omega(t)$, follows from Eq. (4).

The procedure outlined in the previous paragraph allows us to hold fixed ξ_f (and hence the rotation axis and angle) while we adjust parameters in $\chi(t)$. However, it introduces a new difficulty in that the step of solving Eq. (46) can be challenging due to its strongly nonlinear nature. We can simplify this task by expressing $W(t)$ as a function of $\chi(t)$:

$$W(t) = Y(\chi(t)). \tag{49}$$

This allows us to rewrite Eq. (46) as

$$\dot{\chi}^2\left\{1+\lambda^2(\mathrm{Im}[Y'(\chi)/Y(\chi)])^2\sin^2(2\chi)\right\} = \beta^2. \tag{50}$$

Since the term in curly brackets is now purely a function of χ, we may solve this equation by simple integration:

$$\beta t = \int_0^{\chi} d\tilde{\chi}\sqrt{1+\lambda^2(\mathrm{Im}[Y'(\tilde{\chi})/Y(\tilde{\chi})])^2\sin^2(2\tilde{\chi})}. \tag{51}$$

Instead of solving a nonlinear differential equation, the task has now been reduced to performing an ordinary integral and inverting the result. To ensure that ξ_f is quantized, we now need to choose $Y(\chi)$ to be such that it winds an integral number of times around the origin of the complex Y plane as χ evolves from 0 to its final value, χ_f. We can further streamline the determination of $b_z(t)$ by expressing this quantity in terms of $\chi(t)$ using Eq. (50). Explicitly, we find

$$\Omega(t) = \frac{\beta F'(\chi)}{2\lambda \mathrm{Im}[Y'/Y]\sin(2\chi)} - \beta\lambda F(\chi)\mathrm{Im}[Y'/Y]\cos(2\chi), \tag{52}$$

where

$$F(\chi) \equiv \left\{1+\lambda^2(\mathrm{Im}[Y'(\chi)/Y(\chi)])^2\sin^2(2\chi)\right\}^{-1/2}. \tag{53}$$

We can also express $\xi(t)$ as a function of χ:

$$\begin{aligned}\xi(t) &= \lambda\int_0^t dt'\partial_{t'}\arg[W(t')] = \lambda\int_0^t dt'\mathrm{Im}[\dot{W}/W] = \lambda\int_0^t dt'\dot{\chi}(t')\mathrm{Im}[Y'(\chi(t'))/Y(\chi(t'))] \\ &= \lambda\int_0^{\chi(t)} d\tilde{\chi}\mathrm{Im}[Y'(\tilde{\chi})/Y(\tilde{\chi})] = \lambda\mathrm{Im}\log[Y(\chi(t))/Y(0)].\end{aligned} \tag{54}$$

In practice, it turns out to be easier to obtain simpler-looking functions $b_z(t)$ if we work directly with the function

$$\Phi(\chi) \equiv \lambda\mathrm{Im}\log[Y(\chi)/Y(0)], \tag{55}$$

rather than with $Y(\chi)$. The driving field is fully determined by $\Phi(\chi)$:

$$\Omega(\chi) = -\beta\frac{\Phi''(\chi)\sin(2\chi)+4\Phi'(\chi)\cos(2\chi)+2[\Phi'(\chi)]^3\sin^2(2\chi)\cos(2\chi)}{2\left\{1+[\Phi'(\chi)]^2\sin^2(2\chi)\right\}^{3/2}}. \tag{56}$$

To obtain the driving field as a function of time, we need to solve the following equation for χ (see Eq. (51)):

$$\beta t = \int_0^{\chi} d\tilde{\chi}\sqrt{1+[\Phi'(\tilde{\chi})]^2\sin^2(2\tilde{\chi})}. \tag{57}$$

The integrand on the right-hand-side can be identified as the line element obtained from the following metric for a 2-sphere:

$$ds^2 = d\chi^2 + \sin^2(2\chi)d\Phi^2, \tag{58}$$

where we view χ as the azimuthal angle and Φ as the polar angle. This in turn implies that the duration of the pulse,

$$2t_f = \frac{2}{\beta}\int_0^{\chi_f} d\tilde{\chi}\sqrt{1+[\Phi'(\tilde{\chi})]^2\sin^2(2\tilde{\chi})}, \tag{59}$$

is just the length of the curve defined by $\Phi(\chi)$ on the surface of the 2-sphere (times $2/\beta$).

V. DERIVATION OF GENERAL ERROR-CANCELATION CONSTRAINTS

We are now ready to combine all of the ingredients developed in the previous sections to systematically construct rotations that dynamically correct for errors induced by fluctuations in either β or $\Omega(t)$. In order to obtain nontrivial results, it is important that we use the unperturbed quantities $\chi_0(t)$, β_0, and $\xi_0(t)$ in the topological winding formalism developed above.

A. β noise

From the general form of the evolution operator, Eq. (3), we see that in the case of a general single-axis driving field, the first-order error due to $\delta\beta$-noise will vanish only if the following three conditions on the first-order variations hold:

$$\delta_\beta\chi(t_f) = 0, \qquad \delta_\beta\dot{\chi}(t_f) = \frac{\dot{\chi}(t_f)}{\beta_0}\delta\beta, \qquad \delta_\beta\xi(t_f) = 0. \tag{60}$$

Combining the topological winding formalism developed in the previous section with our expression for $\delta_\beta\chi(t)$ in the case of fluctuations in β, Eq. (36), we have

$$\delta_\beta\chi(t) = \delta\beta\frac{2}{\beta_0}\left\{\frac{1}{8}\sin[4\chi_0(t)] + \mathrm{Re}\left[e^{-2i\Phi[\chi_0(t)]}\int_0^{\chi_0(t)} d\tilde{\chi}\sin^2(2\tilde{\chi})e^{2i\Phi(\tilde{\chi})}\right]\right\}. \tag{61}$$

We will make this expression slightly more compact by defining the function

$$G_\beta(\chi) \equiv \int_0^{\chi} d\tilde{\chi}\sin^2(2\tilde{\chi})e^{2i\Phi(\tilde{\chi})}, \tag{62}$$

yielding

$$\delta_\beta\chi(t) = \delta\beta\frac{2}{\beta_0}\left\{\frac{1}{8}\sin[4\chi_0(t)] + \mathrm{Re}\left(e^{-2i\Phi[\chi_0(t)]}G_\beta[\chi_0(t)]\right)\right\}. \tag{63}$$

Introducing the shorthand notation $\delta\chi_f = \delta\chi(t_f)$, etc., the end-point fluctuations are then given by

$$\begin{aligned}\delta_\beta\chi_f &= \delta\beta\frac{2}{\beta_0}\left\{\frac{1}{8}\sin(4\chi_f) + \mathrm{Re}[e^{-2i\Phi(\chi_f)}G_\beta(\chi_f)]\right\},\\ \delta_\beta\dot{\chi}_f &= \delta\beta\frac{\dot{\chi}_f}{\beta_0}\left\{1 + 4\Phi'(\chi_f)\mathrm{Im}[e^{-2i\Phi(\chi_f)}G_\beta(\chi_f)]\right\},\end{aligned} \tag{64}$$

and imposing the first two error-cancelation conditions from Eq. (60) amounts to requiring

$$\begin{aligned}\sin(4\chi_f) + 8\mathrm{Re}[e^{-2i\Phi(\chi_f)}G_\beta(\chi_f)] &= 0,\\ \mathrm{Im}[e^{-2i\Phi(\chi_f)}G_\beta(\chi_f)] &= 0,\end{aligned} \tag{65}$$

which is equivalent to the single complex condition

$$\sin(4\chi_f) + 8e^{-2i\Phi(\chi_f)}G_\beta(\chi_f) = 0. \tag{66}$$

Notice that in these conditions, we have omitted the possibility that $\Phi'(\chi_f) = 0$, which would have automatically guaranteed that $\delta\dot{\chi}_f = (\dot{\chi}_f/\beta_0)\delta\beta$, meaning that we could ignore the second condition in Eq. (65). The reason for this is that when $\Phi'(\chi_f) = 0$ (which implies $\dot{\chi}_f = \beta_0$), we do not completely cancel the first-order error in the final evolution operator because of a square root appearing in its first-order variation stemming from the arcsine part of $\xi_\pm$:

$$\delta_\beta\left(e^{\pm\frac{i}{2}\arcsin(\dot{\chi}_f/\beta)}\right) = \pm i\left(\delta_\beta\dot{\chi}_f - \frac{\dot{\chi}_f}{\beta_0}\delta\beta\right)\frac{e^{\pm\frac{i}{2}\arcsin(\dot{\chi}_f/\beta_0)}}{2\sqrt{\beta_0^2 - \dot{\chi}_f^2}}. \tag{67}$$

If $\dot{\chi}_f = \beta_0$, then the square root in the denominator vanishes, implying that this part of the first-order variation in the evolution will not vanish in general. The only way to guarantee that the first-order error in the evolution operator is completely removed is to *not* impose $\dot{\chi}_f = \beta_0$.

It remains to analyze the third condition in Eq. (60). To calculate $\delta_\beta\xi_f$, we begin with the general expression for $\xi(t)$:

$$\xi(t) = \int_0^t dt'\sqrt{\beta^2 - \dot{\chi}^2}\csc(2\chi). \tag{68}$$

The square root inside the integrand can be expanded as follows:

$$\sqrt{(\beta_0+\delta\beta)^2 - (\dot{\chi}_0+\delta\dot{\chi})^2} \approx \sqrt{\beta_0^2-\dot{\chi}_0^2} - \frac{\dot{\chi}_0\delta\dot{\chi}}{\sqrt{\beta_0^2-\dot{\chi}_0^2}} + \frac{\beta_0\delta\beta}{\sqrt{\beta_0^2-\dot{\chi}_0^2}}, \tag{69}$$

while the remaining factor becomes

$$\csc[2(\chi_0+\delta\chi)] \approx \csc(2\chi_0) - 2\delta\chi\cot(2\chi_0)\csc(2\chi_0). \tag{70}$$

We therefore have

$$\delta\xi(t) = -2\int_0^t dt'\sqrt{\beta_0^2-\dot{\chi}_0^2}\cot(2\chi_0)\csc(2\chi_0)\delta\chi - \int_0^t dt'\dot{\chi}_0\frac{\csc(2\chi_0)}{\sqrt{\beta_0^2-\dot{\chi}_0^2}}\delta\dot{\chi} + \beta_0\delta\beta\int_0^t dt'\frac{\csc(2\chi_0)}{\sqrt{\beta_0^2-\dot{\chi}_0^2}}. \tag{71}$$

In the case of β-noise, we substitute the expressions for $\delta_\beta\chi(t)$ and $\delta_\beta\dot{\chi}(t)$ from Eq. (63) into Eq. (71) to obtain

$$\begin{aligned}\delta_\beta\xi(t) &= -\delta\beta\frac{4}{\beta_0}\int_0^t dt'\sqrt{\beta_0^2-\dot{\chi}_0^2}\cot(2\chi_0)\csc(2\chi_0)\left\{\frac{1}{8}\sin(4\chi_0) + \mathrm{Re}[e^{-2i\Phi(\chi_0)}G_\beta(\chi_0)]\right\}\\ &\quad -\delta\beta\frac{1}{\beta_0}\int_0^t dt'\dot{\chi}_0^2\frac{\csc(2\chi_0)}{\sqrt{\beta_0^2-\dot{\chi}_0^2}}\left\{1+4\Phi'(\chi_0)\mathrm{Im}[e^{-2i\Phi(\chi_0)}G_\beta(\chi_0)]\right\} + \beta_0\delta\beta\int_0^t dt'\frac{\csc(2\chi_0)}{\sqrt{\beta_0^2-\dot{\chi}_0^2}}.\end{aligned} \tag{72}$$

Using the relation $\sqrt{\beta_0^2-\dot{\chi}_0^2} = \dot{\chi}_0\Phi'(\chi_0)\sin(2\chi_0)$, we can eliminate all of the square roots:

$$\begin{aligned}\delta_\beta\xi(t) &= -\delta\beta\frac{4}{\beta_0}\int_0^t dt'\dot{\chi}_0\Phi'(\chi_0)\cot(2\chi_0)\left\{\frac{1}{8}\sin(4\chi_0) + \mathrm{Re}[e^{-2i\Phi(\chi_0)}G_\beta(\chi_0)]\right\}\\ &\quad -\delta\beta\frac{1}{\beta_0}\int_0^t dt'\dot{\chi}_0\frac{\csc^2(2\chi_0)}{\Phi'(\chi_0)}\left\{1+4\Phi'(\chi_0)\mathrm{Im}[e^{-2i\Phi(\chi_0)}G_\beta(\chi_0)]\right\} + \beta_0\delta\beta\int_0^t dt'\frac{\csc^2(2\chi_0)}{\dot{\chi}_0\Phi'(\chi_0)}.\end{aligned} \tag{73}$$

With the help of the relation

$$\dot{\chi}_0 = \frac{\beta_0}{\sqrt{1+[\Phi'(\chi_0)]^2\sin^2(2\chi_0)}}, \tag{74}$$

we can write each term as an integral over χ:

$$\begin{aligned}\delta_\beta\xi(t) &= -\delta\beta\frac{4}{\beta_0}\int_0^{\chi_0(t)} d\chi\Phi'(\chi)\cot(2\chi)\left\{\frac{1}{8}\sin(4\chi)+\mathrm{Re}[e^{-2i\Phi(\chi)}G_\beta(\chi)]\right\} \\ &\quad -\delta\beta\frac{1}{\beta_0}\int_0^{\chi_0(t)} d\chi\frac{\csc^2(2\chi)}{\Phi'(\chi)}\left\{1+4\Phi'(\chi)\mathrm{Im}[e^{-2i\Phi(\chi)}G_\beta(\chi)]\right\}+\delta\beta\frac{1}{\beta_0}\int_0^{\chi_0(t)} d\chi\frac{\csc^2(2\chi)}{\Phi'(\chi)}\left\{1+[\Phi'(\chi)]^2\sin^2(2\chi)\right\} \\ &= \delta\beta\frac{1}{\beta_0}\int_0^{\chi_0(t)} d\chi\Phi'(\chi)\left\{\sin^2(2\chi)-4\cot(2\chi)\mathrm{Re}[e^{-2i\Phi(\chi)}G_\beta(\chi)]\right\}-\delta\beta\frac{4}{\beta_0}\int_0^{\chi_0(t)} d\chi\csc^2(2\chi)\mathrm{Im}[e^{-2i\Phi(\chi)}G_\beta(\chi)]\end{aligned} \tag{75}$$

This result can be simplified dramatically if we perform an integration by parts on the last term:

$$\int_0^{\chi_0} d\chi\csc^2(2\chi)\mathrm{Im}[e^{-2i\Phi(\chi)}G_\beta(\chi)] = -\frac{1}{2}\cot(2\chi)\mathrm{Im}[e^{-2i\Phi(\chi)}G_\beta(\chi)]\Big|_0^{\chi_0}-\int_0^{\chi_0} d\chi\cot(2\chi)\Phi'(\chi)\mathrm{Re}[e^{-2i\Phi(\chi)}G_\beta(\chi)]. \tag{76}$$

The second term here precisely cancels a similar-looking term in Eq. (75), leaving us with

$$\delta_\beta\xi(t)=\frac{\delta\beta}{\beta_0}\int_0^{\chi_0(t)} d\chi\Phi'(\chi)\sin^2(2\chi)+2\frac{\delta\beta}{\beta_0}\cot[2\chi_0(t)]\mathrm{Im}\left\{e^{-2i\Phi[\chi_0(t)]}G_\beta[\chi_0(t)]\right\}, \tag{77}$$

where we have also used that

$$\lim_{\chi\to 0}\cot(2\chi)\mathrm{Im}[e^{-2i\Phi(\chi)}G_\beta(\chi)]=0. \tag{78}$$

The requirement that $\delta_\beta\xi_f=0$ then yields the following condition:

$$\delta_\beta\xi_f=\frac{\delta\beta}{\beta_0}\int_0^{\chi_f} d\chi\Phi'(\chi)\sin^2(2\chi)+2\frac{\delta\beta}{\beta_0}\cot(2\chi_f)\mathrm{Im}\left\{e^{-2i\Phi(\chi_f)}G_\beta(\chi_f)\right\}=0. \tag{79}$$

However, the second term in this condition is already required to vanish according to the additional constraints we have derived earlier (see Eq. (65)), so the first term must also vanish independently:

$$\int_0^{\chi_f} d\chi\Phi'(\chi)\sin^2(2\chi)=0. \tag{80}$$

In summary, the following two constraints are necessary and sufficient for ensuring that the leading-order error in the evolution operator due to β-noise vanishes identically:

$$\begin{aligned}\sin(4\chi_f)+8e^{-2i\Phi(\chi_f)}\int_0^{\chi_f} d\chi\sin^2(2\chi)e^{2i\Phi(\chi)} &= 0, \\ \int_0^{\chi_f} d\chi\Phi'(\chi)\sin^2(2\chi) &= 0.\end{aligned} \tag{81}$$

B. $\Omega(t)$ noise

From the general form of the evolution operator, Eq. (3), we see that in the case of a general single-axis driving field, the first-order error due to Ω-noise will vanish only if the following three conditions on the first-order variations hold:

$$\delta_\epsilon\chi(t_f)=0,\qquad \delta_\epsilon\dot{\chi}(t_f)=0,\qquad \delta_\epsilon\xi(t_f)=0. \tag{82}$$

In Sec. III, we found the fluctations in $\chi(t)$ caused by fluctuations in $\Omega(t)$, Eq. (44). Using the results of Sec. IV, this can be rewritten as

$$\delta_\epsilon\chi(t) = \delta\epsilon\frac{1}{2\beta}\left\{\beta k\cos(2\Phi[\chi_0(t)])+\mathrm{Im}\left[e^{-2i\Phi[\chi_0(t)]}\int_0^{\chi_0(t)} d\tilde{\chi}\sin(2\tilde{\chi})\frac{\tilde{g}(\tilde{\chi})}{F(\tilde{\chi})}e^{2i\Phi(\tilde{\chi})}\right]\right\}, \tag{83}$$

where we have defined $\tilde{g}$ such that $g(t) = \tilde{g}(\chi_0(t))$. Defining the function

$$G_\epsilon(\chi) \equiv \int_0^\chi d\tilde{\chi} \sin(2\tilde{\chi}) \frac{\tilde{g}(\tilde{\chi})}{F(\tilde{\chi})} e^{2i\Phi(\tilde{\chi})}, \tag{84}$$

and setting $k = 0$ as is appropriate for fluctuations that originate from the control field $\Omega(t)$, this becomes

$$\begin{aligned} \delta_\epsilon \chi(t) &= \delta\epsilon \frac{1}{2\beta} \text{Im} \left[e^{-2i\Phi[\chi_0(t)]} G_\epsilon[\chi_0(t)] \right], \\ \delta_\epsilon \dot{\chi}(t) &= -\delta\epsilon \frac{1}{\beta} \dot{\chi}_0(t) \Phi'[\chi_0(t)] \text{Re} \left[e^{-2i\Phi[\chi_0(t)]} G_\epsilon[\chi_0(t)] \right]. \end{aligned} \tag{85}$$

As in the case of β-noise, we again want to avoid setting $\Phi'(\chi_f) = 0$ since this will lead to an incomplete cancelation of the first-order error. We are then left with the following condition necessary for canceling the error:

$$G_\epsilon(\chi_f) = 0. \tag{86}$$

We now turn to the third condition from Eq. (82). For driving field noise, we may again use the first-order variation of $\xi(t)$, Eq. (71), but now drop the term involving $\delta\beta$:

$$\delta_\epsilon \xi(t) = -2 \int_0^t dt' \sqrt{\beta_0^2 - \dot{\chi}_0^2} \cot(2\chi_0) \csc(2\chi_0) \delta_\epsilon \chi - \int_0^t dt' \dot{\chi}_0 \frac{\csc(2\chi_0)}{\sqrt{\beta_0^2 - \dot{\chi}_0^2}} \delta_\epsilon \dot{\chi}. \tag{87}$$

Using the expressions for $\delta_\epsilon \chi(t)$ and $\delta_\epsilon \dot{\chi}(t)$ given earlier in Eq. (85), this becomes

$$\delta_\epsilon \xi(t) = -\frac{\delta\epsilon}{\beta_0} \int_0^t dt' \sqrt{\beta_0^2 - \dot{\chi}_0^2} \cot(2\chi_0) \csc(2\chi_0) \text{Im}[e^{-2i\Phi(\chi_0)} G_\epsilon(\chi_0)] + \frac{\delta\epsilon}{\beta_0} \int_0^t dt' \dot{\chi}_0^2 \frac{\csc(2\chi_0)}{\sqrt{\beta_0^2 - \dot{\chi}_0^2}} \Phi'(\chi_0) \text{Re}[e^{-2i\Phi(\chi_0)} G_\epsilon(\chi_0)]. \tag{88}$$

We next remove the square roots and turn the integrations over t' into integrations over χ using $\sqrt{\beta_0^2 - \dot{\chi}_0^2} = \dot{\chi}_0 \Phi'(\chi_0) \sin(2\chi_0)$:

$$\delta_\epsilon \xi(t) = -\frac{\delta\epsilon}{\beta_0} \int_0^{\chi_0(t)} d\chi \Phi'(\chi) \cot(2\chi) \text{Im}[e^{-2i\Phi(\chi)} G_\epsilon(\chi)] + \frac{\delta\epsilon}{\beta_0} \int_0^{\chi_0(t)} d\chi \csc^2(2\chi) \text{Re}[e^{-2i\Phi(\chi)} G_\epsilon(\chi)]. \tag{89}$$

Integrating the second term by parts,

$$\begin{aligned} \int_0^{\chi_0} d\chi \csc^2(2\chi) \text{Re}[e^{-2i\Phi(\chi)} G_\epsilon(\chi)] &= -\frac{1}{2} \cot(2\chi) \text{Re}[e^{-2i\Phi(\chi)} G_\epsilon(\chi)] \Big|_0^{\chi_0} + \frac{1}{2} \int_0^{\chi_0} d\chi \cos(2\chi) \frac{\tilde{g}(\chi)}{F(\chi)} \\ &\quad + \int_0^{\chi_0} d\chi \cot(2\chi) \Phi'(\chi) \text{Im}[e^{-2i\Phi(\chi)} G_\epsilon(\chi)], \end{aligned} \tag{90}$$

we find that the third term here cancels the first term in Eq. (89), leaving

$$\delta_\epsilon \xi(t) = \frac{\delta\epsilon}{2\beta_0} \int_0^{\chi_0(t)} d\chi \cos(2\chi) \frac{\tilde{g}(\chi)}{F(\chi)} - \frac{\delta\epsilon}{2\beta_0} \cot[2\chi_0(t)] \text{Re} \left\{ e^{-2i\Phi[\chi_0(t)]} G_\epsilon[\chi_0(t)] \right\}. \tag{91}$$

The end-point variation is then

$$\delta_\epsilon \xi_f = \frac{\delta\epsilon}{2\beta_0} \int_0^{\chi_f} d\chi \cos(2\chi) \frac{\tilde{g}(\chi)}{F(\chi)} - \frac{\delta\epsilon}{2\beta_0} \cot(2\chi_f) \text{Re}[e^{-2i\Phi(\chi_f)} G_\epsilon(\chi_f)]. \tag{92}$$

When we impose the earlier condition from Eq. (86), the second term vanishes, in turn requiring that the first term also vanishes independently. In summary, the following two constraints are necessary and sufficient for ensuring that the leading-order error in the evolution operator due to Ω-noise vanishes identically:

$$\begin{aligned} \int_0^{\chi_f} d\chi \sin(2\chi) \tilde{g}(\chi) e^{2i\Phi(\chi)} \sqrt{1 + [\Phi'(\chi)]^2 \sin^2(2\chi)} &= 0, \\ \int_0^{\chi_f} d\chi \cos(2\chi) \tilde{g}(\chi) \sqrt{1 + [\Phi'(\chi)]^2 \sin^2(2\chi)} &= 0. \end{aligned} \tag{93}$$

VI. CONSTRUCTING ROBUST CONTROL FIELDS

Here, we collect all the preceding results and summarize the general approach to constructing robust control fields. These control fields are completely determined by a function $\Phi(\chi)$ which we are free to choose. We can make the initial evolution operator the identity matrix by imposing $\Phi(0) = 0$ and $\Phi'(0) = 0$. Choosing $\eta = -1$ in Eq. (3) and focusing on the case of single-axis driving, we find that the target evolution operator is determined by the value of Φ and its first derivative at a chosen final value of χ, which we denote by χ_f:

$$\begin{aligned} U_{target} &= \begin{pmatrix} \cos\chi_f e^{i\xi_-(t_f)} & -i\sin\chi_f e^{-i\xi_+(t_f)} \\ -i\sin\chi_f e^{i\xi_+(t_f)} & \cos\chi_f e^{-i\xi_-(t_f)} \end{pmatrix}, \\ \xi_\pm(t_f) &= \Phi(\chi_f) \mp \frac{1}{2}\mathrm{arcsec}\left(\sqrt{1+[\Phi'(\chi_f)]^2\sin^2(2\chi_f)}\right). \end{aligned} \tag{94}$$

A driving field $\Omega(t)$ which generates this target evolution via the Hamiltonian $H = \Omega(t)\sigma_z + \beta\sigma_x$ can be found by performing the following integration

$$\beta t = \int_0^{\chi_0} d\chi\sqrt{1+[\Phi'(\chi)]^2\sin^2(2\chi)}, \tag{95}$$

inverting the result to obtain $\chi_0(t)$, and plugging this into

$$\Omega(\chi) = -\beta\frac{\Phi''(\chi)\sin(2\chi) + 4\Phi'(\chi)\cos(2\chi) + 2[\Phi'(\chi)]^3\sin^2(2\chi)\cos(2\chi)}{2\left\{1+[\Phi'(\chi)]^2\sin^2(2\chi)\right\}^{3/2}}. \tag{96}$$

The most general way to ensure that the resulting pulse has finite duration is to require that the numerator of the expression for $\Omega(\chi)$, Eq. (96), vanishes at $\chi = \chi_f$. We should think of this condition as a condition on the final value of the second derivative of $\Phi(\chi)$:

$$\Phi''(\chi_f) = -4\Phi'(\chi_f)\cot(2\chi_f) - 2[\Phi'(\chi_f)]^3\sin(2\chi_f)\cos(2\chi_f). \tag{97}$$

Viewing the condition this way is appropriate since we have already fixed χ_f and $\Phi'(\chi_f)$ in accordance with our desired U_{target}. We can further ensure that the resulting pulse amplitude has zero slope at the beginning and end of the pulse by imposing that the third derivative of $\Phi(\chi)$ vanishes at $\chi = 0$ and $\chi = \chi_f$. This will force the pulse to have flattened tails, which in turn can reduce sensitivity to timing errors.

If we want the leading-order errors in the evolution to vanish, then we must constrain not only the boundary conditions of $\Phi(\chi)$ but its full behavior from $\chi = 0$ to $\chi = \chi_f$ as well. In the case of β-noise, $\Phi(\chi)$ must satisfy

$$\begin{aligned} \sin(4\chi_f) + 8e^{-2i\Phi(\chi_f)}\int_0^{\chi_f} d\chi\sin^2(2\chi)e^{2i\Phi(\chi)} &= 0, \\ \int_0^{\chi_f} d\chi\Phi'(\chi)\sin^2(2\chi) &= 0. \end{aligned} \tag{98}$$

whereas in the case of $\Omega(t)$-noise, it must satisfy

$$\begin{aligned} \int_0^{\chi_f} d\chi\sin(2\chi)\tilde{g}(\chi)e^{2i\Phi(\chi)}\sqrt{1+[\Phi'(\chi)]^2\sin^2(2\chi)} &= 0, \\ \int_0^{\chi_f} d\chi\cos(2\chi)\tilde{g}(\chi)\sqrt{1+[\Phi'(\chi)]^2\sin^2(2\chi)} &= 0, \end{aligned} \tag{99}$$

where the function $\tilde{g}(\chi)$ is determined by the precise nature of the noise one wishes to consider: $\delta\Omega = \tilde{g}(\chi_0(t))\delta\epsilon$. For example, pulse amplitude fluctuations are described by choosing $\tilde{g}(\chi) = \Omega(\chi)$. If one wishes to cancel errors due to both types of noise simultaneously, then $\Phi(\chi)$ must satisfy both sets of constraints.

The fact that the arbitrary time-dependence in the Ω-noise fluctuation carries all the way through the calculation and leads to the appearance of the arbitrary function $\tilde{g}(\chi)$ in Eq. (99) suggests that the analysis could be extended to the case of non-static noise. In particular, one could consider performing a Gaussian path integral average of $\tilde{g}$, weighted by a nontrivial noise power spectrum. Since $\tilde{g}$ appears linearly inside a single integration in Eq. (99), it is likely that this average could be performed analytically using standard Gaussian integration techniques. This analysis will be carried out elsewhere.

A straightforward way to solve the above constraints on $\Phi(\chi)$, either Eqs. (98) or Eqs. (99), is to start with an ansatz for $\Phi(\chi)$ of the form

$$\Phi(\chi) = a_1\chi^2 + a_2\chi^3 + f(\chi), \tag{100}$$

where a_1 and a_2 are constants and f is such that $f(0) = f'(0) = f'(\chi_f) = f''(\chi_f) = 0$. We can set any desired U_{target} by choosing χ_f, a_1, and a_2 appropriately. We may then include additional parameters in $f(\chi)$ and tune these until the error-cancelation constraints are satisfied. For example, if we choose

$$f(\chi) = a_3 \sin^3(\pi\chi/\chi_f) + a_4 \sin^3(2\pi\chi/\chi_f), \tag{101}$$

then we may freely tune a_3 and a_4 without changing U_{target}. This ansatz is used to construct the robust driving fields shown in Fig. 1 of the main text for the case of a driving field subject to noise in its amplitude.

Interestingly, it is also possible to solve the β-noise cancelation constraints analytically for a certain set of target rotations. In particular, we can solve the first constraint in Eq. (98) when $\chi_f = n\pi/4$ for some integer n by choosing $\Phi(\chi)$ to have the general form

$$\Phi(\chi) = [4\chi - \sin(4\chi) + \lambda\zeta(4\chi - \sin(4\chi))]/n, \tag{102}$$

where $\zeta(\theta + n\pi/2) = \zeta(\theta)$ is any periodic function with period $n\pi/2$, and λ is an arbitrary real parameter. We can automatically solve the second constraint in Eq. (98) for any non-constant $\zeta(\theta)$ by setting λ to

$$\lambda^{-1} = \frac{2}{3\pi n}\int_0^{n\pi} d\theta \sin\theta\zeta(\theta - \sin\theta). \tag{103}$$

This solution gives rise to a class of target evolutions determined by n and $\lambda\zeta'(n\pi)$; in particular, the phases in the evolution operator are

$$\xi_\pm(t_f) = n\pi \mp \frac{1}{2}\text{arcsec}\left(\sqrt{1 + \frac{64}{n^2}\sin^6\left(\frac{n\pi}{2}\right)[1 + \lambda\zeta'(n\pi)]^2}\right). \tag{104}$$

If n is an even integer but not a multiple of 4, then U_{target} reduces to a π-rotation about the x axis. If n is a multiple of 4, then U_{target} is proportional to the identity. In this case, the control field implements a smooth-pulse version of dynamical decoupling.

The fact that the β-noise constraints can be solved analytically greatly simplifies the problem in the case where one wishes to cancel both types of noise simultaneously. To do so, one could start with a more general ansatz for Φ:

$$\Phi(\chi) = \frac{1}{n}\left[4\chi - \sin(4\chi) + \sum_k \lambda_k\zeta_k(4\chi - \sin(4\chi))\right], \tag{105}$$

where all the $\zeta_k(\theta)$ are periodic functions of period $n\pi/2$. This ansatz automatically solves the β-noise constraints provided the λ_k satisfy one linear constraint coming from the second condition in Eq. (98). The remaining λ_k can then be tuned until the Ω-noise constraints are also satisfied.

VII. ANTISYMMETRIC PULSES

If χ is an odd function, $\chi(-t) = -\chi(t)$, which implies that $\Omega(-t) = -\Omega(t)$, then the total evolution operator which evolves the qubit from $t = -t_f$ to $t = t_f$ takes a particularly simple form which leads to fewer error-cancelation constraints that need to be satisfied. The antisymmetry of $\chi(t)$ immediately implies that the phases in the evolution operator enjoy the following property under time reversal:

$$\xi_\pm(-t_f) = \xi_\pm(t_f). \tag{106}$$

Using this relation, it can be shown that the full evolution is described by the operator

$$U_{tot} = U(t_f)\sigma_z U(t_f)^\dagger \sigma_z, \tag{107}$$

where $U(t_f)$ describes the evolution from $t = 0$ to $t = t_f$. Eq. (107) leads to the expressions

$$\begin{aligned} U_{tot,11} &= \cos(2\chi_f), \\ U_{tot,12} &= i\eta\sin(2\chi_f))e^{i\xi_-(t_f) - i\xi_+(t_f)}, \end{aligned} \tag{108}$$

from which we extract the following total evolution operator components for antisymmetric pulses:

$$\begin{aligned}
\mathrm{Re}[U_{tot,11}] &= \cos(2\chi_f), \\
\mathrm{Im}[U_{tot,11}] &= 0, \\
\mathrm{Re}[U_{tot,12}] &= \sqrt{1-\frac{\dot{\chi}_f^2}{\beta^2}}\sin(2\chi_f), \\
\mathrm{Im}[U_{tot,12}] &= -\frac{\dot{\chi}_f}{\beta}\sin(2\chi_f).
\end{aligned} \tag{109}$$

This evolution operator corresponds to rotations about axes in the xy plane. The axis and angle of rotation can be expressed in terms of χ_f and $\Phi'(\chi_f)$ according to

$$\phi = 4\chi_f, \qquad \cos\theta = \frac{1}{\sqrt{1+[\Phi'(\chi_f)]^2\sin^2(2\chi_f)}}, \tag{110}$$

where θ is the angle between the axis of rotation (which lies in the xy plane) and the x-axis, and ϕ is the angle of rotation. Thus finding a $\Phi(\chi)$ that implements a desired evolution amounts to imposing the following condition on the derivative of $\Phi(\chi)$ at $\chi_f = \phi/4$:

$$\Phi'(\phi/4) = \tan\theta\csc(\phi/2). \tag{111}$$

Because U_{tot} does not depend on $\xi(t_f)$ in the case of an antisymmetric pulse, we do not need to impose the error-cancelation constraint associated with fluctuations in $\xi(t_f)$. This means that we may ignore the second constraints in Eqs. (98) and (99), and we need only solve the first constraints in each set to obtain robust antisymmetric control fields.

VIII. UNIVERSAL SET OF ROTATIONS

To perform an arbitrary single qubit rotation, it suffices to be able to rotate by an arbitrary angle around one chosen axis, plus being able to do a π rotation around an axis $\pi/4$ (45 degrees) apart from that chosen axis. The latter gate serves a similar role as the Hadamard gate which turns any rotation around one axis into a rotation with the same angle around an axis perpendicular to it.

In this section we present the parameters required to perform a set of rotations around an axis in the x-y plane lying at an angle θ from the x-axis, namely

$$R(\theta,\phi) = \exp\left[-i(\sin\theta\sigma_y+\cos\theta\sigma_x)\frac{\phi}{2}\right]. \tag{112}$$

For each of the two cases discussed in the main text (pulses correcting driving field errors and $\delta\beta$ errors), we give pulses that implement rotations $R(5\pi/12,\pi)$ and $R(\pi/6,\phi)$ for a range of ϕ. The two rotation axes are $\pi/4$ apart as required. Due to the strong nonlinearity of the problem, it is not practical to scan over the entire $[0,2\pi)$ range of ϕ continuously; we therefore demonstrate a set of rotations with angles between π and 3π with step 0.2π. Since our method is completely general and can take as many parameters as needed, it is straightforward to find the pulses for any single-qubit rotation.

A. Pulses correcting driving field errors

For pulses correcting driving field errors, we make use of the ansatz

$$\Phi(\chi) = a_1\sin^2(a_2\chi) + a_3\sin^3\left(\frac{4\pi\chi}{\phi}\right) + a_4\sin^3\left(\frac{8\pi\chi}{\phi}\right). \tag{113}$$

Parameters for the set of rotations discussed above are provided in Table I.

$R(\theta,\phi)$	a_1	a_2	a_3	a_4
$R(5\pi/12,\pi)$	1.24402	3.00000	2.01146	1.26906
$R(\pi/6,\pi)$	-0.577350	1.00000	2.29863	1.01756
$R(\pi/6,1.2\pi)$	0.273558	2.33333	2.37161	1.17764
$R(\pi/6,1.4\pi)$	-0.237992	3.35888	2.30057	1.28314
$R(\pi/6,1.6\pi)$	1.72135	1.41514	2.70120	1.50434
$R(\pi/6,1.8\pi)$	9.96178	1.16814	0.829187	1.87861
I	1.00000	1.00000	0	1.99924
$R(\pi/6,2.2\pi)$	0.317272	7.94004	1.75940	3.25893
$R(\pi/6,2.4\pi)$	-1.46213	1.54756	-1.14086	1.33871
$R(\pi/6,2.6\pi)$	2.84789	-0.676288	1.91457	-0.791508
$R(\pi/6,2.8\pi)$	1.78210	-0.568079	-0.451979	0.271430

TABLE I: Parameters of pulses correcting driving field errors for a set of rotations.

B. Pulses correcting $\delta\beta$-noise

For pulses correcting $\delta\beta$-noise, we use the ansatz

$$\Phi(\chi) = a_1 \sin^2(a_2\chi) + a_3 \sin^3\left(\frac{4N_3\pi\chi}{\phi}\right) + a_4 \sin^3\left[\frac{\pi(\phi-4\chi)}{\phi}\right] + a_5 \sin^3\left[\frac{\pi}{4}\chi(\phi-4\chi)\right], \tag{114}$$

where N_3 must be an integer. Parameters for the set of rotations discussed above are provided in Table II.

$R(\theta,\phi)$	a_1	a_2	a_3	a_4	a_5	N_3
$R(5\pi/12,\pi)$	1.24402	3.00000	-0.464647	1.42922	10.4704	1
$R(\pi/6,\pi)$	-0.577350	1.00000	-1.41354	0.480222	30.4015	1
$R(\pi/6,1.2\pi)$	0.273558	2.33333	0.727786	2.62177	-3.27545	1
$R(\pi/6,1.4\pi)$	-0.237992	3.35888	2.12682	4.02077	-6.92089	1
$R(\pi/6,1.6\pi)$	1.72135	1.41514	-0.725379	0.172736	-0.885146	2
$R(\pi/6,1.8\pi)$	9.96178	1.16814	-0.108777	-1.01910	-0.978449	1
$R(\pi/6,2.2\pi)$	0.317272	7.94004	-3.12414	0.803819	2.94582	2
$R(\pi/6,2.4\pi)$	5.45961	0.770847	2.74485	8.04491	4.95240	1
$R(\pi/6,2.6\pi)$	2.84789	-0.676289	4.24974	5.15883	4.27094	1
$R(\pi/6,2.8\pi)$	-0.598662	1.19958	-1.14903	-0.24894	0.665241	1

TABLE II: Parameters of pulses correcting $\delta\beta$-noise for a set of rotations.

IX. CONSTRUCTION OF ORDINARY PULSES AND DEFINITION OF FIDELITY

In Figs. 2 and 3 of the main text, we verify that the first-order error in the evolution operator has been canceled by comparing the infidelity of the designed pulse with that of a square pulse sequence that implements the same target evolution but which has not been designed to combat errors. In the case of single-axis driving, we can systematically construct a square pulse sequence that generates any target evolution operator using the following general form involving two identical square pulses:

$$U_{target} = R_{\beta_0}(0;t_c)R_{\beta_0}(\beta_0;\tau)R_{\beta_0}(0;t_b)R_{\beta_0}(\beta;\tau)R_{\beta_0}(0;t_a), \tag{115}$$

where $R_\beta(\Omega;t) \equiv e^{-it(\Omega\sigma_z+\beta\sigma_x)}$ and $\tau = \pi/(2\sqrt{2}\beta_0)$. Given U_{target}, we can solve this equation numerically to obtain the parameters t_a, t_b, t_c. In Figs. 2c and 3c of the main text, we use the definition of infidelity as in Ref. [5]:

$$\text{infidelity} = \frac{1}{2} - \frac{1}{12}\sum_{x,y,z} \text{Tr}\left\{U_{target}\sigma_j U^\dagger_{target} U(t_f)\sigma_j U^\dagger(t_f)\right\}. \tag{116}$$

Here, $U(t_f)$ is the actual evolution operator including the errors to all orders. In the case of β-noise for example, the evolution corresponding to the uncorrected pulses is given simply by

$$U(t_f) = R_{\beta_0+\delta\beta}(0;t_c)R_{\beta_0+\delta\beta}(\beta_0;\tau)R_{\beta_0+\delta\beta}(0;t_b)R_{\beta_0+\delta\beta}(\beta;\tau)R_{\beta_0+\delta\beta}(0;t_a), \tag{117}$$

while $U(t_f)$ for the designed pulses is computed by solving the Schrödinger equation numerically with Hamiltonian $H = \Omega(t)\sigma_z + (\beta_0 + \delta\beta)\sigma_x$ for each value of the error strength, $\delta\beta$.

[1] E. Barnes, Phys. Rev. A **88**, 013818 (2013).
[2] J. R. Petta, A. C. Johnson, J. M. Taylor, E. A. Laird, A. Yacoby, M. D. Lukin, C. M. Marcus, M. P. Hanson, and A. C. Gossard, Science **309**, 2180 (2005).
[3] J. J. Pla, K. Y. Tan, J. P. Dehollain, W. H. Lim, J. J. L. Morton, F. A. Zwanenburg, D. N. Jamieson, A. S. Dzurak, and A. Morello, Nature **496**, 334 (2013).
[4] E. Barnes and S. Das Sarma, Phys. Rev. Lett. **109**, 060401 (2012).
[5] M. D. Bowdrey, D. K. L. Oi, A. J. Short, K. Banaszek, and J. A. Jones, Phys. Lett. A **294**, 258 (2002).
